# Supplementary material for: Acute Exacerbation of COPD
Source: J Educ Teach Emerg Med. 2023 Apr 30;8(2):S35–61. doi: 10.21980/J8V070 (PMC10332676; doi:10.21980/J8V070)

## Slide 1
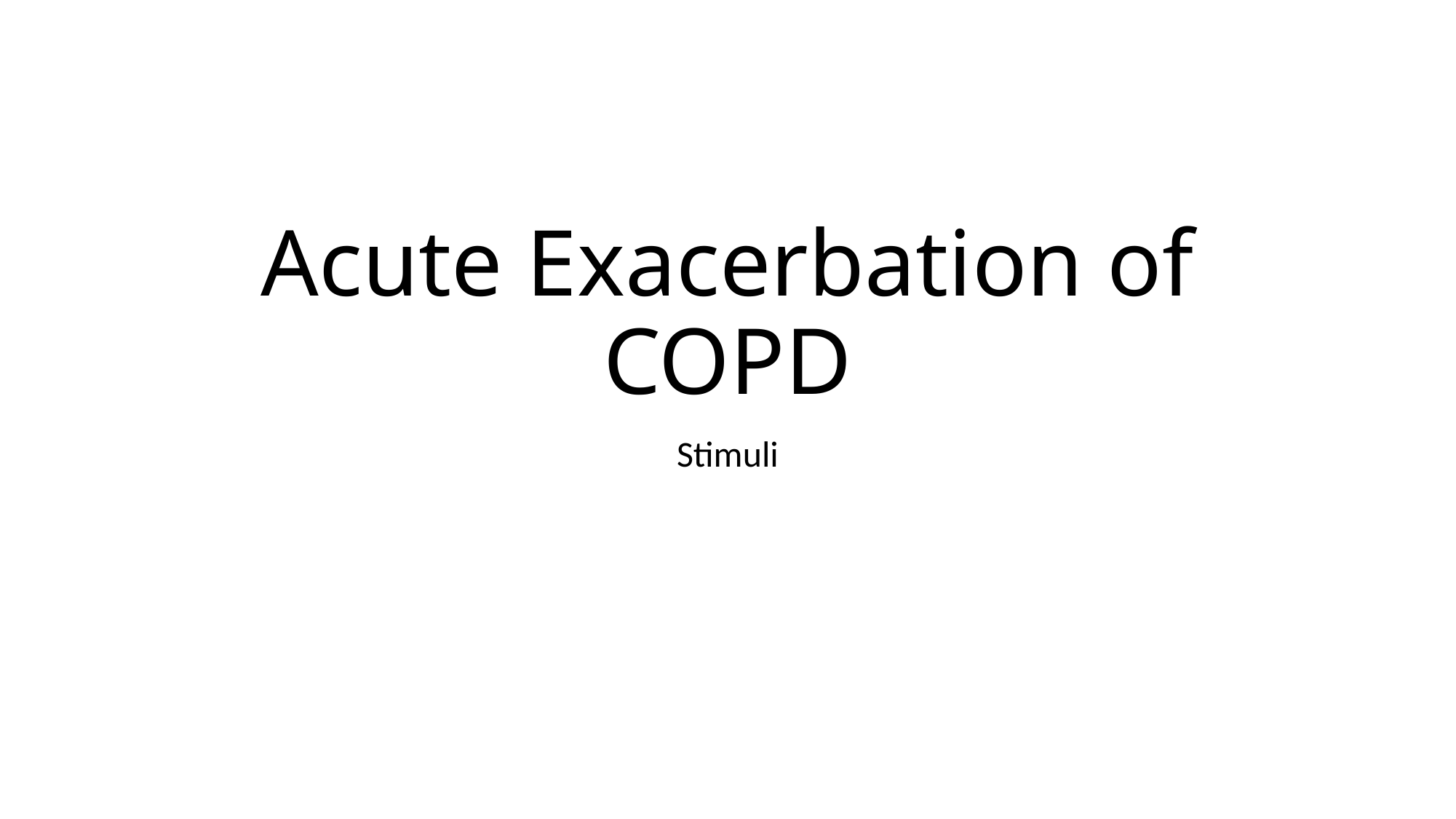

# Acute Exacerbation of COPD
Stimuli

## Slide 2
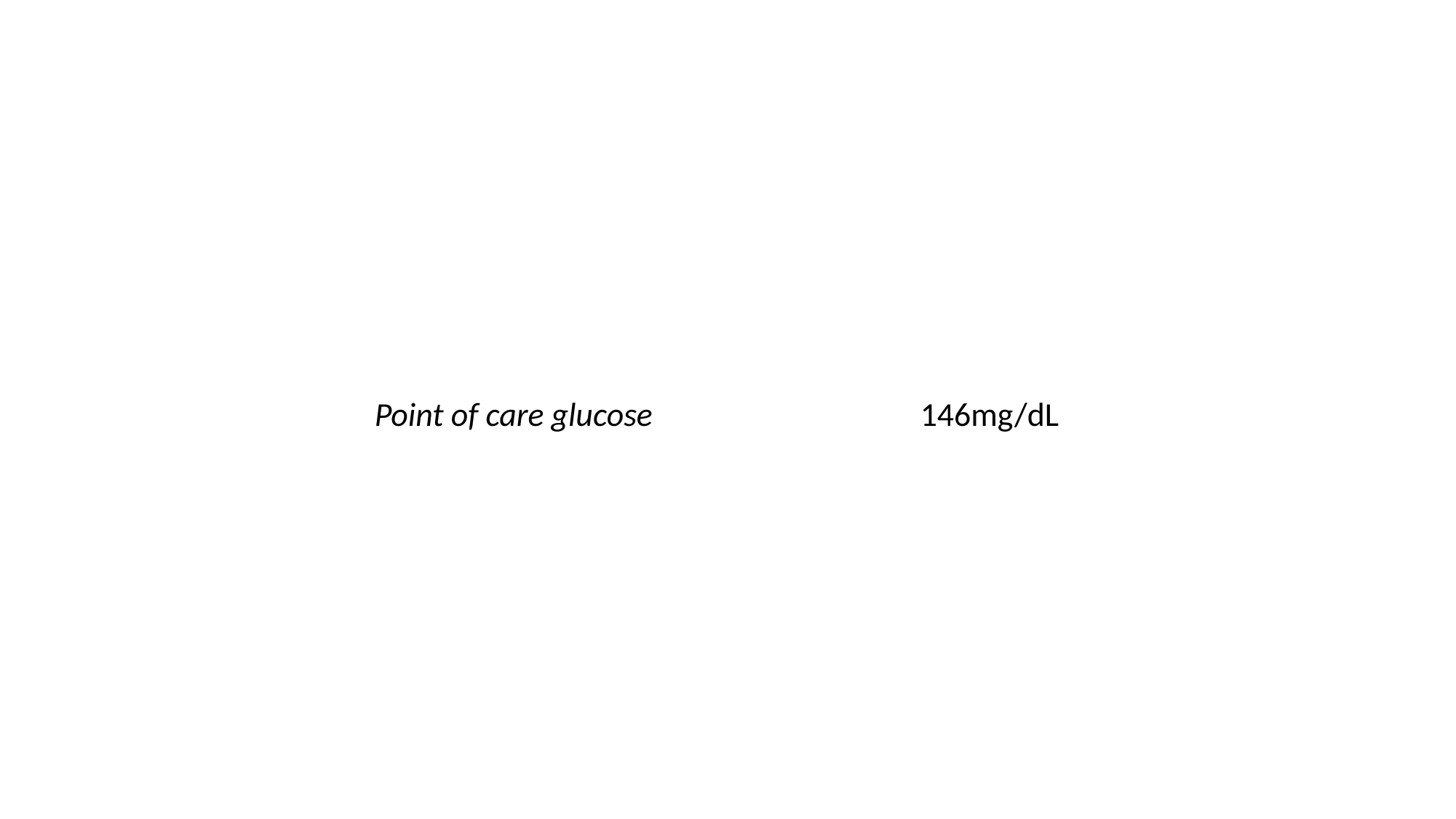

Point of care glucose			146mg/dL

## Slide 3
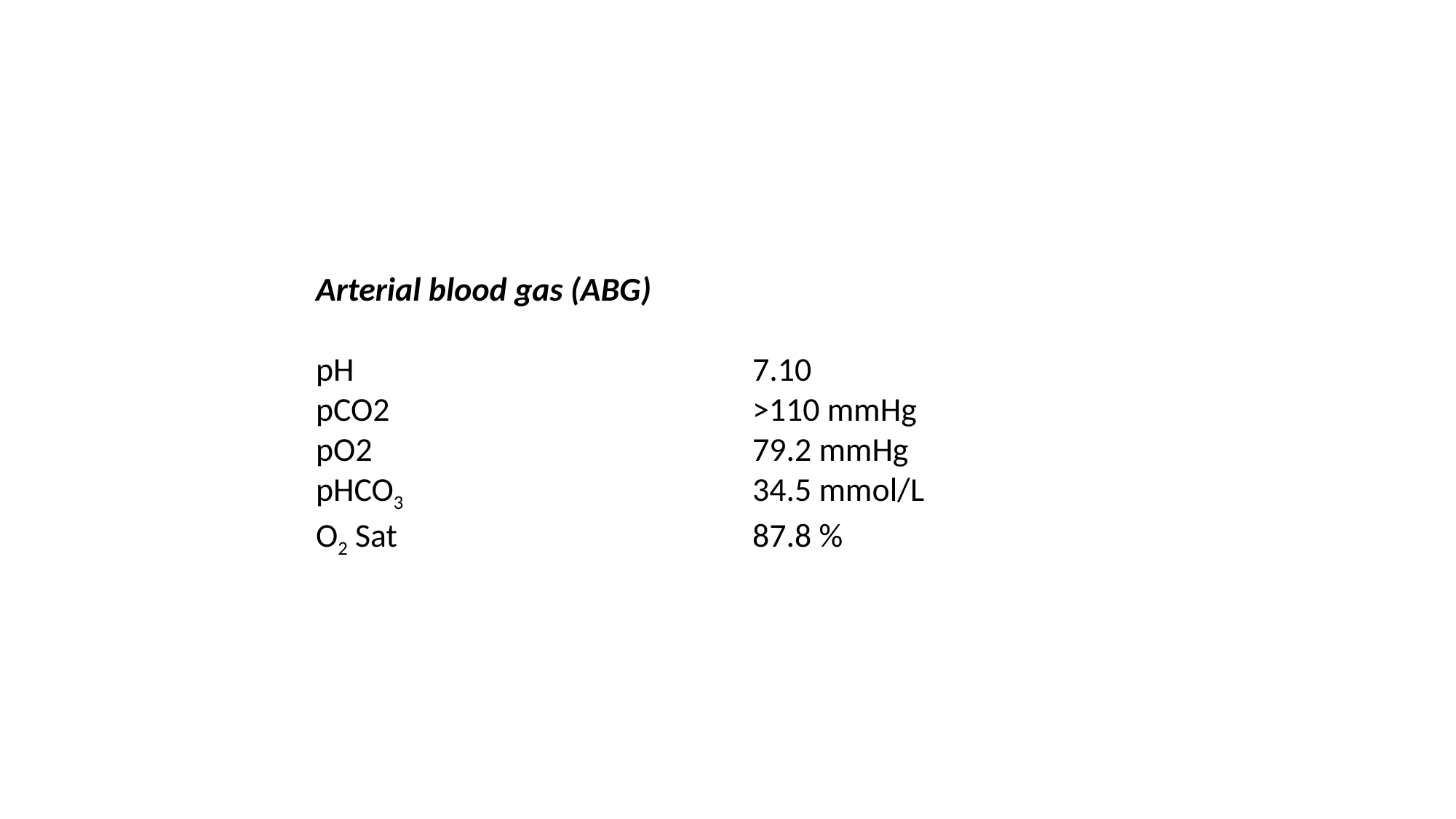

Arterial blood gas (ABG)
pH 				7.10
pCO2 				>110 mmHg
pO2 				79.2 mmHg
pHCO3 				34.5 mmol/L
O2 Sat 				87.8 %

## Slide 4
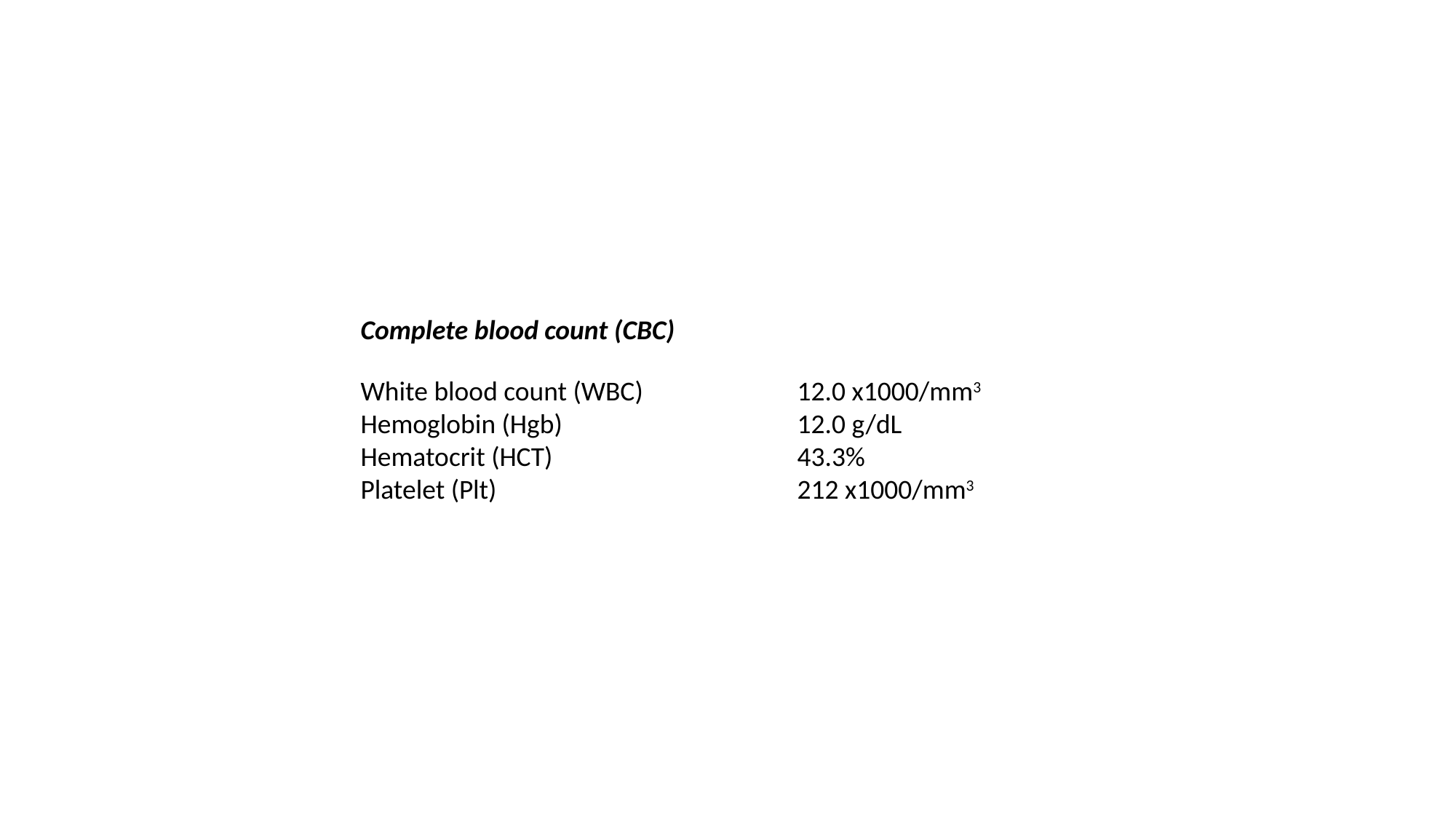

Complete blood count (CBC)
White blood count (WBC) 		12.0 x1000/mm3
Hemoglobin (Hgb)			12.0 g/dL
Hematocrit (HCT)			43.3%
Platelet (Plt) 			212 x1000/mm3

## Slide 5
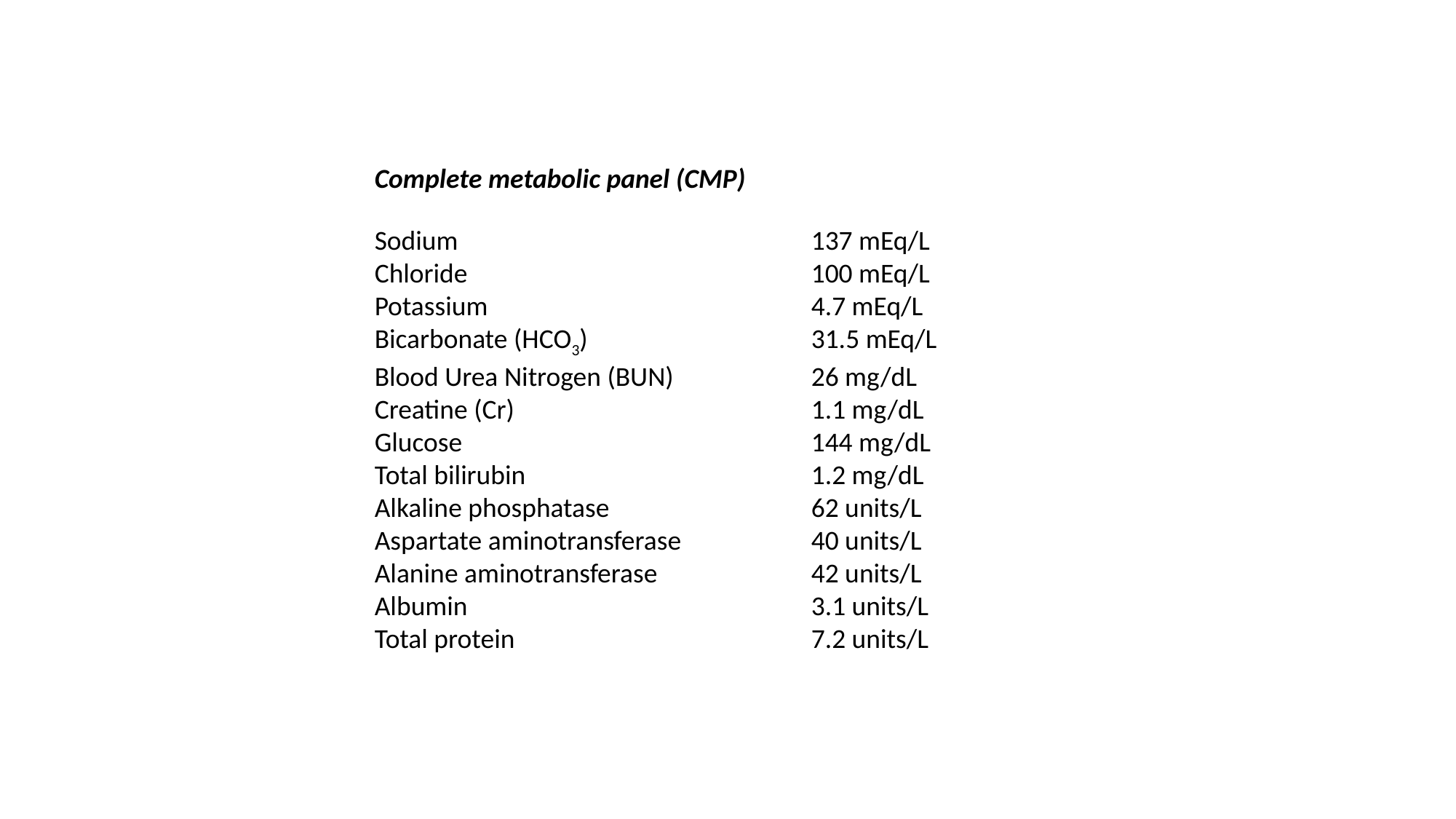

Complete metabolic panel (CMP)
Sodium 				137 mEq/L
Chloride 				100 mEq/L
Potassium			4.7 mEq/L
Bicarbonate (HCO3)			31.5 mEq/L
Blood Urea Nitrogen (BUN)		26 mg/dL
Creatine (Cr)		 	1.1 mg/dL
Glucose 				144 mg/dL
Total bilirubin			1.2 mg/dL
Alkaline phosphatase		62 units/L
Aspartate aminotransferase		40 units/L
Alanine aminotransferase		42 units/L
Albumin				3.1 units/L
Total protein			7.2 units/L

## Slide 6
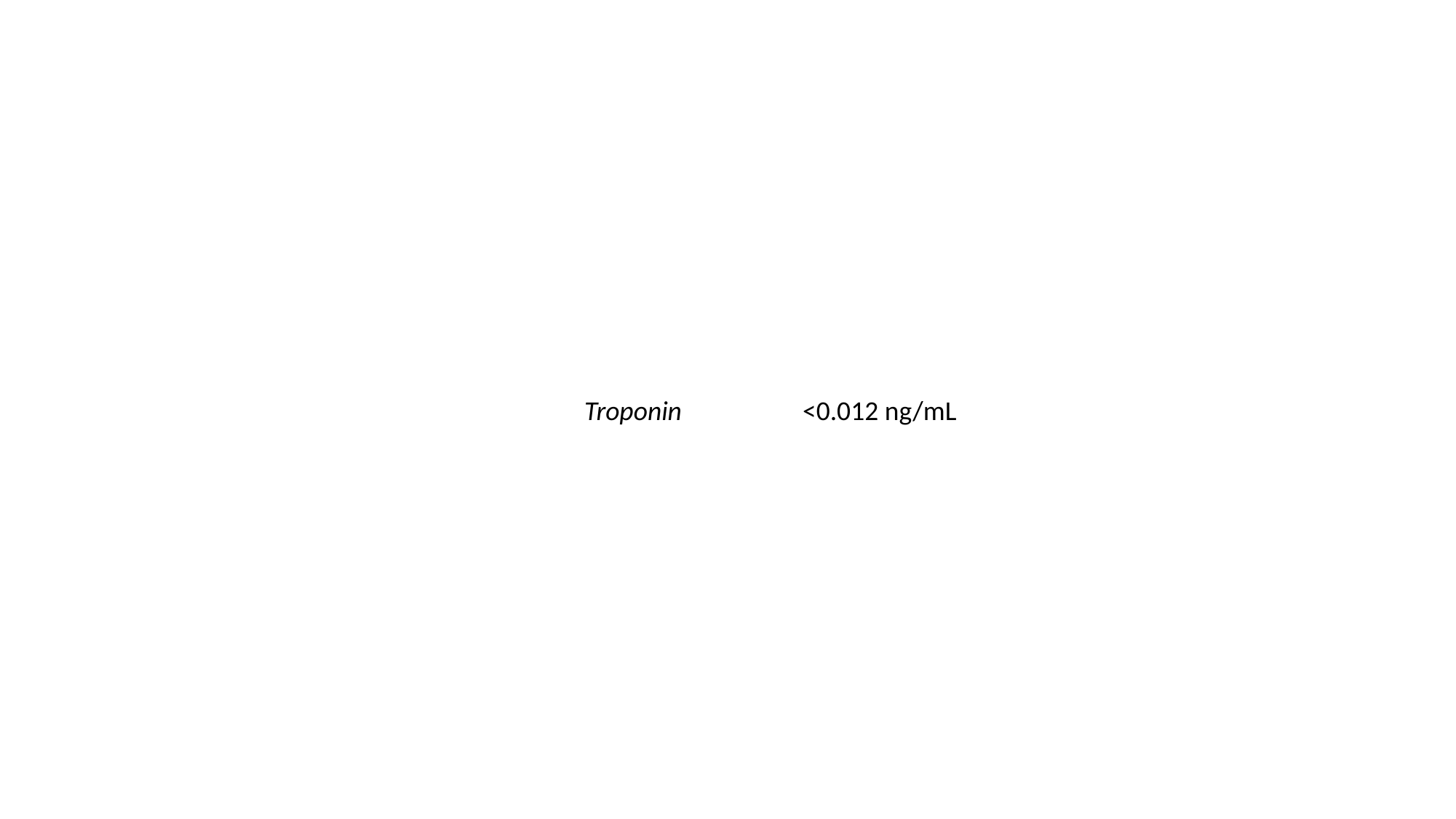

Troponin		<0.012 ng/mL

## Slide 7
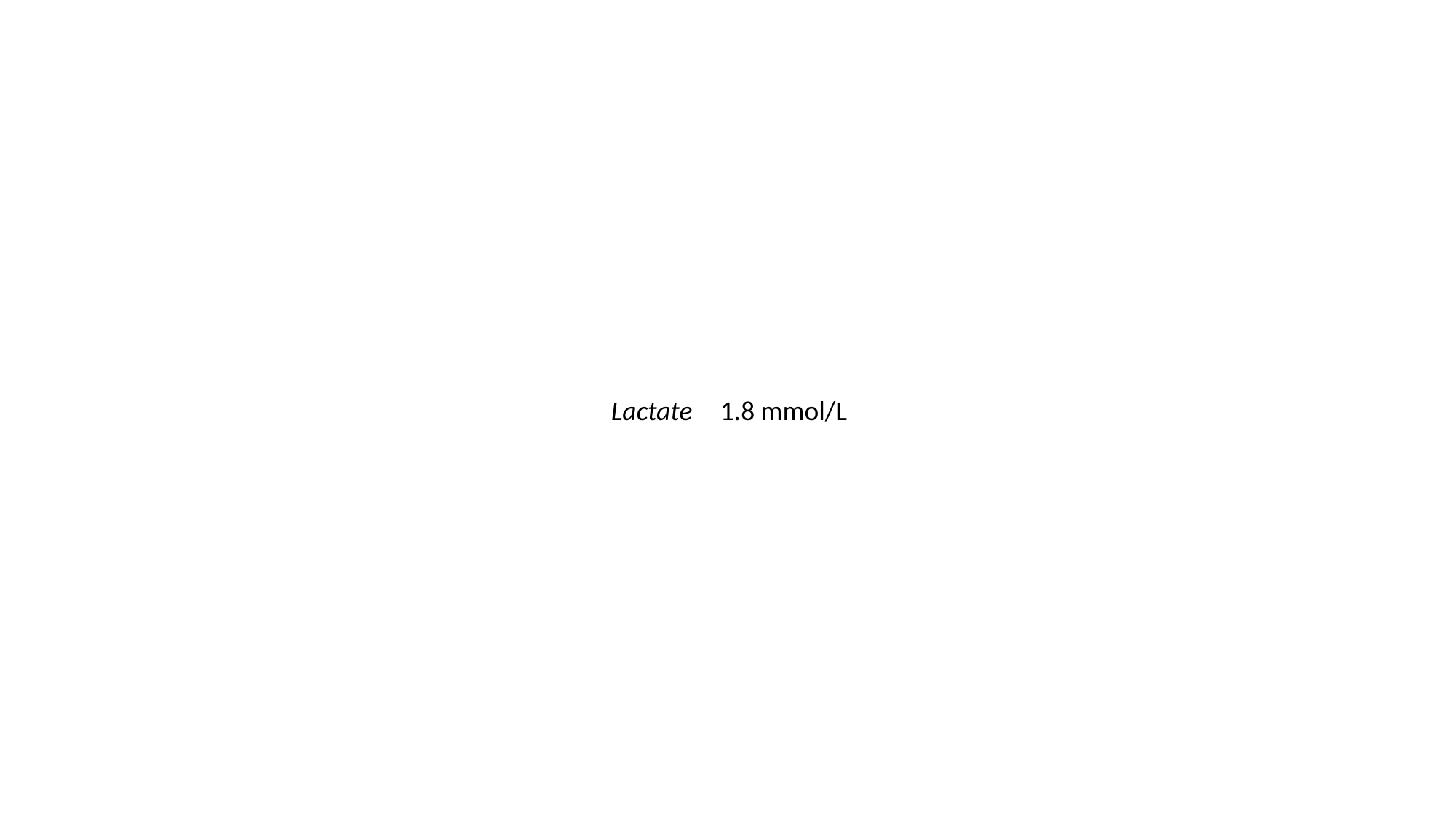

Lactate	1.8 mmol/L

## Slide 8
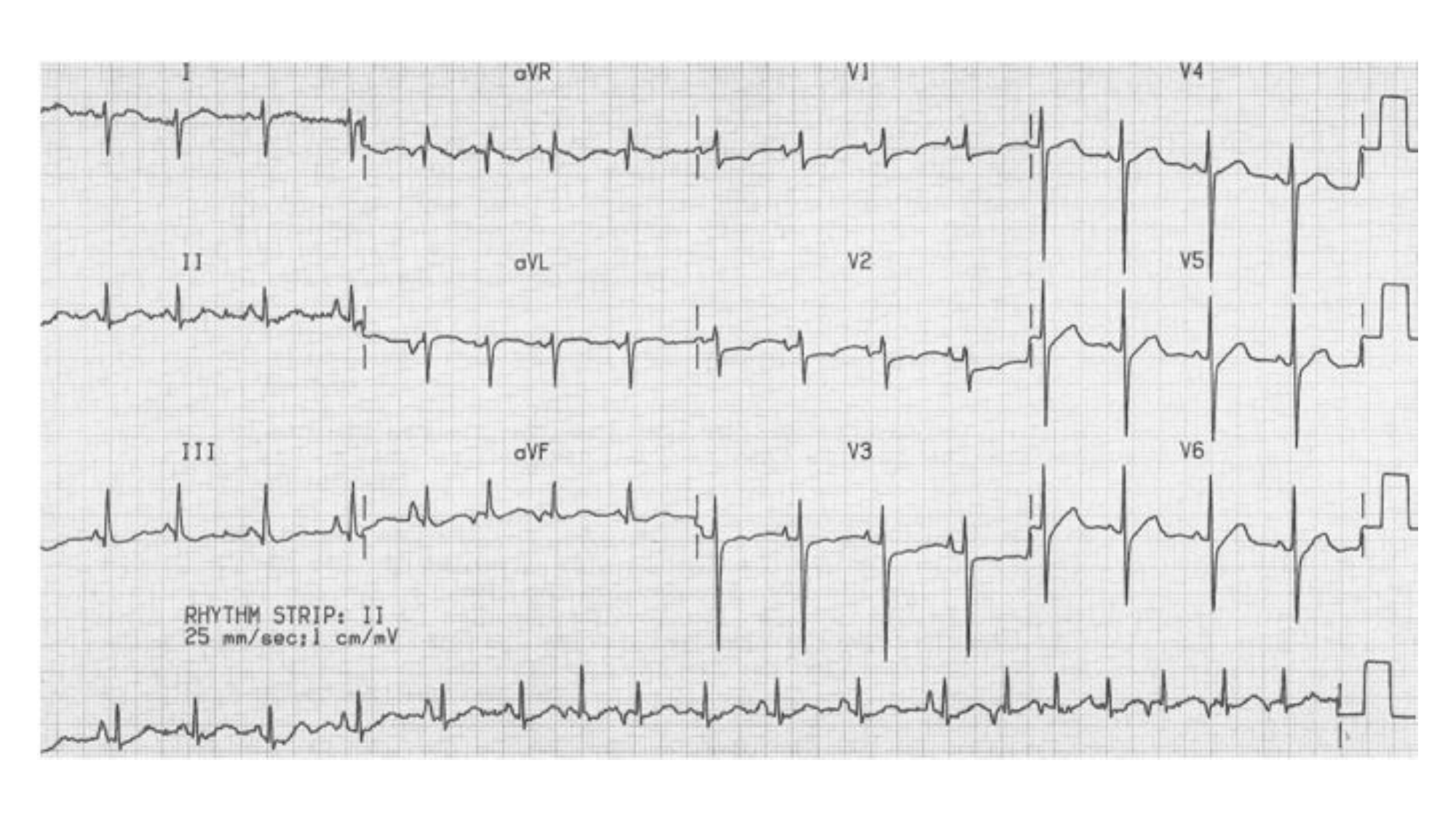

## Slide 9
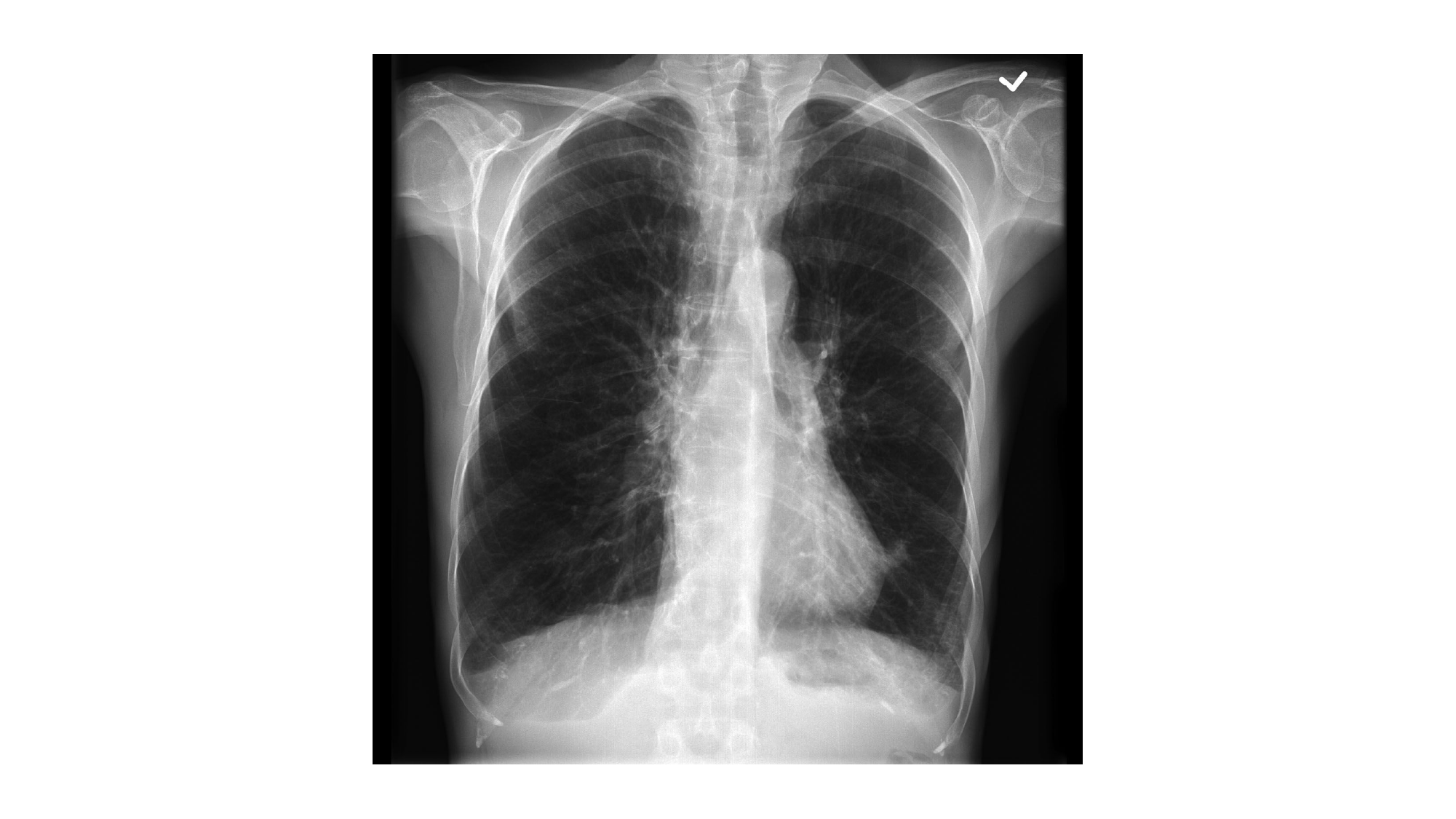

Supplement: Supplementary file 1 [file JETem-8-2-S35-Supp1.pptx]
